# When Pinocchio's nose does not grow: belief regarding lie-detectability modulates production of deception

**DOI:** 10.3389/fnhum.2013.00016

**Published:** 2013-02-04

**Authors:** Kamila E. Sip, David Carmel, Jennifer L. Marchant, Jian Li, Predrag Petrovic, Andreas Roepstorff, William B. McGregor, Christopher D. Frith

**Affiliations:** ^1^Center of Functionally Integrative Neuroscience, Aarhus University HospitalAarhus, Denmark; ^2^Department of Aesthetics and Communication - Linguistics, Aarhus UniversityAarhus, Denmark; ^3^Department of Psychology, Rutgers University - NewarkNJ, USA; ^4^Department of Psychology, The University of EdinburghEdinburgh, UK; ^5^Institute of Cognitive Neuroscience, University College LondonLondon, UK; ^6^Wellcome Trust Centre for Neuroimaging, University College LondonLondon, UK; ^7^Department of Psychology, IDG/McGovern Institute for Brain Research Peking UniversityBeijing, China; ^8^Department of Clinical Neuroscience, Karolinska Institutet Stockholm, A Medical UniversityStockholm, Sweden

**Keywords:** mock-crime, deception, beliefs, lie-detection, fMRI

## Abstract

Does the brain activity underlying the production of deception differ depending on whether or not one believes their deception can be detected? To address this question, we had participants commit a mock theft in a laboratory setting, and then interrogated them while they underwent functional MRI (fMRI) scanning. Crucially, during some parts of the interrogation participants believed a lie-detector was activated, whereas in other parts they were told it was switched-off. We were thus able to examine the neural activity associated with the contrast between producing true vs. false claims, as well as the independent contrast between believing that deception could and could not be detected. We found increased activation in the right amygdala and inferior frontal gyrus (IFG), as well as the left posterior cingulate cortex (PCC), during the production of false (compared to true) claims. Importantly, there was a significant interaction between the effects of deception and belief in the left temporal pole and right hippocampus/parahippocampal gyrus, where activity increased during the production of deception when participants believed their false claims could be detected, but not when they believed the lie-detector was switched-off. As these regions are associated with binding socially complex perceptual input and memory retrieval, we conclude that producing deceptive behavior in a context in which one believes this deception can be detected is associated with a cognitively taxing effort to reconcile contradictions between one's actions and recollections.

## Introduction

Deception is inherently social. Deceptive behavior involves not only the creation of a representation that is at odds with physical reality, but also the manipulation of another person's beliefs in a particular context (Sip et al., 2008a). This, in turn, means that deceivers must hold a belief about whether their deception is likely to be detected because a high likelihood of detection may lead to anxiety, altering the deceiver's emotional state and arousal level. Although several recent studies have attempted to elucidate the neural underpinnings of producing (e.g., Abe et al., 2007; Baumgartner et al., 2009; Kozel et al., 2009; Sip et al., 2010) and detecting (Grèzes et al., 2004, 2006) deceptive behavior, the role of beliefs about the detectability of deception remains poorly understood.

Behavioral research has shown that neither deceivers nor truthful people respond in the same way to all situations, as their behavior depends on their emotional state (Ekman and Friesen, 1969; Ekman, 1992), the complexity of what is said (Vrij, 2000; Vrij et al., 2001), and their need to control the impression they make on others (Vrij, 1993). From a behavioral standpoint, therefore, there is no diagnostic cue that serves as a unique indication of deception (Vrij, 2004; Vrij et al., 2007; Vrij, 2008). This may be due to the complex nature of the demands that deceptive behavior places on the deceiver: it requires a series of conjectures about the deceived person's knowledge, the gap between this knowledge and the truth, the feasible manipulations this gap leaves room for, and the chances of getting caught. Deception is thus a sophisticated activity, involving a host of cognitive processes including memory, reasoning, and theory of mind. Furthermore, producing deception is emotionally taxing, and causes anxiety and physiological arousal that require effortful self-regulation (e.g., Abe et al., 2007; Baumgartner et al., 2009).

The multi-faceted act of attempting to deceive is therefore likely to require the concerted activity of several neural mechanisms, with activity in different, widely distributed brain regions mediating the various processes underlying deceptive behavior. Recently, a great deal of interest has centered on neuroimaging to test whether this technology could prove to be a useful and reliable tool for lie-detection [for review see Greely and Illes (2007); Sip et al. (2008a,b)]. Several physiological (e.g., Bell et al., 2008; Gamer et al., 2010, 2012) and functional MRI (fMRI) studies (see e.g., Kozel et al., 2005, 2009; Mohamed et al., 2006) of mock-crimes have investigated the neural correlates of information inhibition and suppression that are associated with deceptive behavior. This previous research, however, has focused almost entirely on comparing deceptive vs. truthful behavior, neglecting the potential effects of participants' belief in the efficacy of lie-detection, and how such belief may modulate the neural activity underlying deception. Peoples' beliefs about whether or not their deception can be detected may affect activity in all brain regions that are involved in the production of this behavior. Alternatively, such belief may only modulate activity in a subset of these regions—for example, the belief that deception may be detected might alter activity in regions whose activity mediates the emotional aspects of deceptive behavior, but not those mediating aspects related to memory and reasoning. Clarifying this issue has both theoretical implications for understanding the systems underlying deception, and practical implications for the use of neuroimaging in forensic contexts.

In the current study we used a mock-theft paradigm to investigate whether people's beliefs about lie-detectability affect the brain activity that underlies the production of deception. Instead of focusing primarily on comparing the neural activity evoked by participants' false and true claims, we investigated whether people's beliefs regarding whether or not their false responses can be detected affect the brain activity underlying the production of these responses. By analogy to the well-known story of Pinocchio's growing nose, we asked: would Pinocchio's nose only grow when he *believed* his lies could be detected?

Subjective beliefs about the world and other people underlie most social and socio-economic decisions (e.g., Frith and Frith, 2003). Specifically, our beliefs and expectations modulate our emotional and physiological states, the way we interact with others, and how we make and evaluate choices (e.g., Pollina et al., 2004; Petrovic et al., 2005; De Martino et al., 2006; Mobbs et al., 2006; Sip et al., 2010, 2012). Deception is an instance of belief manipulation, and is likely to rely on the deceiver's own beliefs.

Previous studies of deception have found increased activation in the amygdala—a region known to be involved in the processing of emotional information—when participants produce (Abe et al., 2007; Baumgartner et al., 2009) and detect deception (Grèzes et al., 2004, 2006). Additionally, several other regions known to mediate cognitive processes involving memory and reasoning, such as the inferior frontal gyrus (IFG), the anterior and posterior cingulate cortex (ACC, PCC, respectively) have been associated with producing false responses (e.g., Spence et al., 2001; Ganis et al., 2003; Langleben et al., 2005; Nuñez et al., 2005; Gamer et al., 2007; Sip et al., 2012). The amygdala, in particular, seems to be a likely candidate for modulation by production of deception and belief due to its central role in emotional processing and its ubiquitous involvement in belief-related tasks (Grèzes et al., 2004, 2006; Abe et al., 2007; Baumgartner et al., 2009). Grèzes et al. (2004, 2006) conducted two studies on non-verbal deception in which participants either judged whether a third party deceived them (2004), or witnessed deception which they were not the target of themselves (2006). Increased activation in the amygdala was found in both studies only when participants detected that they were being deceived by a third party. More recently, other groups found amygdala activation to be associated with breaking a previously made promise (Abe et al., 2007; Baumgartner et al., 2009). Taken together, these studies suggest that the amygdala plays an important role in processing deception, regardless of whether one is personally engaged in producing it or is a target of deceit.

Inhibiting a choice of a risky option has been shown to be associated with risk evaluation and risk aversion in cases where no deception was involved (Aron et al., 2004; Christopoulos et al., 2009). The IFG has been implicated in production of deception where participants needed to inhibit their true responses (Langleben et al., 2005; Gamer et al., 2007). In a recent study by Sip et al. (2012), activation in the right IFG was observed when participants were deciding whether or not to produce a falsehood. This activation occurred regardless of which response, true or false, was made, which suggests that the IFG integrates contextual information about a risky choice rather than the value of a claim itself. It remains unknown, however, whether this region mediates any belief-related activity.

Here, we had participants commit a mock-crime (stealing a gadget they were motivated to keep) and then undergo a realistic interrogation, designed to induce increased anxiety, while undergoing fMRI scanning. Importantly, we manipulated their belief about the detectability of their deception by notifying them that a (fictitious) lie-detector was either active or inactive during different parts of the interrogation. We expected this manipulation to modulate activity in the network of brain regions previously associated with producing deception—the amygdala, IFG, ACC, and PCC. Our main goal was to find out whether belief would alter activity in all these areas, only some of them, or an entirely separate set of neural regions.

## Materials and methods

### Participants

Nineteenth healthy, right-handed participants with no reported neurological or psychiatric disorders, and from diverse social and professional backgrounds, took part in the experiment. Data from two participants were removed from the analysis. One admitted to stealing an object in the first few questions; the other fell asleep during the functional scans. The remaining 17 participants (7 females) were between 20 and 45 years old. Participants gave written informed consent to take part in the study, which was approved by the Joint Ethics Committee of the National Hospital for Neurology and Neuroscience (UCL NHS Trust) and Institute of Neurology (UCL).

### Stimuli and procedure

Upon arrival at the laboratory, each participant was given both written and verbal instructions. Participants were told that they would steal an item and that afterwards, as they were interrogated in the scanner while connected to a lie-detector, their brain activity would be monitored. Unknown to the participants, the “lie-detector” was not real, and comprised two mock electrodes and a finger grip to imitate a polygraph test.

Two rooms were used in the mock-theft stage. The rooms were marked “red” and “blue” by pieces of appropriately colored paper placed on the inside and outside of each door. Each room contained typical office furniture and items, among which were a pair of earphones and a USB memory stick. The earphones and memory sticks were placed out of immediate view, in specific locations known to the researchers.

Each participant was escorted by the experimenter (author KES) to the corridor outside the red and blue rooms. Participants were informed that they had the right to refrain from taking part in the study, if it conflicted with their morals, and they would still be paid for participation. No participant took this option.

The participants were asked to enter each room and search it carefully in order to locate the earphones and the USB memory stick. They were asked to select one room and “steal” a single object from it. Participants could enter the rooms as many times as they wanted, but were asked to go into each room at least once in order to become familiar with both rooms and locate all the objects. After taking an object, they put it into an opaque bag provided by the experimenter, and hid it in a locker before going into the scanner.

In the scanner control room, the participant met the interrogator, who was introduced as an expert in the field of criminal investigation, with a specialty in polygraph tests (the interrogator was actually either author DC or PP, who are not, in fact, such specialists; one was assigned to each participant randomly). Before entering the control room they were told the interrogator did not know whether or not they had stolen anything, but only that they had been inside both rooms and had searched them. They were also told that if, by the end of the interrogation, the interrogator could not tell whether they had taken an object, then they would get to keep the object they took (in fact, the interrogators were aware that all participants had taken an object, and half of the participants were selected at random and allowed to keep the stolen object). The interrogator explained the procedure of the interrogation, and presented the equipment that would ostensibly be used to measure skin conductance responses (or GSR, for galvanic skin responses, the acronym used during the interrogation). To illustrate “typical” skin conductance readings, the interrogator presented computer-generated graphs to the participants. These graphs were unrelated to real polygraph readings; one showed a relatively smooth line and, according to the interrogator, indicated “telling the truth,” while the other was very spiky and indicated “lying.” The aim of this presentation was to persuade the participants that the lie-detection device works reliably.

Participants were told that the “lie-detector” would enable the interrogator to discriminate between honest and deceptive responses. However, it would only be turned on for half the time during each scanning session, and they would be informed when this was happening.

**Figure 1:**
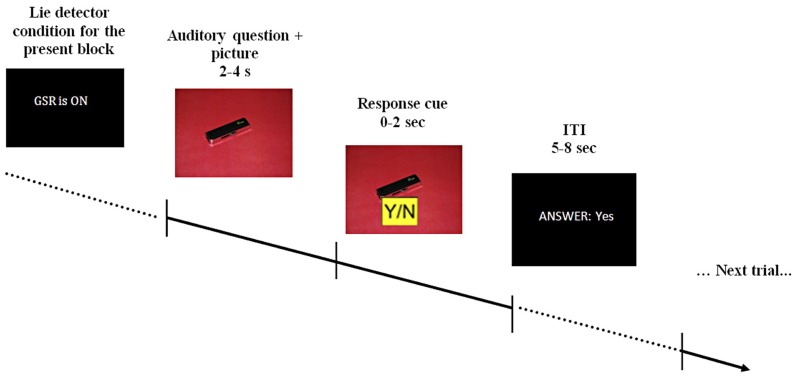
**A schematic example of the stimulus display.** At the beginning of each block participants were told that the lie-detector (represented by the acronym GSR, for galvanic skin response) was either on or off. During the interrogation, pre-recorded auditory questions were read out over earphones, accompanied by appropriate visual presentations (question presentation took 2–4 s). After the question was completed, a response cue appeared on the screen for 2 s, during which participants had to provide a response. The response cue (0–2 s) was randomly assigned on each trial (Y/N or N/Y) to prevent participants from pressing only one button as a default response. Participants' response (which could be either “yes,” “no,” “no response” if no response was given within the allotted time or “wrong button” if a button without an assigned meaning was pressed) was displayed on the screen for the duration of the 5–8 s inter-trial interval (ITI).

The questions used during scanning were pre-recorded and played in a randomized order. Pre-recorded comments, such as “I see you're finding this difficult,” were also used to maintain a realistic atmosphere. Depending on its content, each question was accompanied by a picture of either the red or blue room, or by a picture of one of the objects on an appropriately colored background (see Figure 1). Participants were asked to answer the questions by pressing keys marked yes/no on a response pad (two-specific keys on a four-key pad), as well as to mouth their response with a pre-specified noise—[mm]/əməm/for “no” and [mhm]/əmhm/for “yes”—to verify they were attending to the task. Participants were informed both auditory and with written text each time the “lie-detector” was supposedly turned on or off.

Participants were not explicitly instructed to produce false statements, but merely motivated to try and keep the object they took. The questions used during the interrogation fell into several categories. A set of 12 personal questions, such as “Is your name John?” or “Are you British?” was used to acquaint participants with the procedure; they were told that such questions were used to establish a “baseline” for their skin conductance responses. A further set of 11 general questions allowed true responses to non-incriminating aspects of participants' behavior, e.g., “*Did you go into the Red-room?*” which would always elicit a true response of “yes,” because all the participants were asked to perform the same set of actions. Together, the personal and general questions helped establish a realistic atmosphere.

The crucial part of the interrogation consisted of 35 theft-related questions, divided between truth- and falsehood-eliciting based on each participant's choice of stolen object: (1) theft-related falsehood-eliciting questions (14 related to the theft of an object that the participant actually took); and (2) 21 theft-related truth-eliciting questions (related to the theft of an object that the participant did not take). The nature of a specific theft-related question (truth- or falsehood-eliciting), as well as the number of questions of each type, was determined by context. For example, a question such as “*Did you take earphones from the Red-Room?*” would be a theft-related falsehood-eliciting question, to which the participant would respond “no,” if they took the object from that room. The same question would be a theft-related truth-eliciting question (again evoking the response “no”), if the participant took the earphones from the other room. The experiment thus had a 2 (belief: lie-detector on, lie-detector off) × 2 (behavior: true, false) factorial design. Each participant was scanned twice, with each of the two scanning sessions divided into one half with the lie-detector “on” and the other with the lie-detector “off.” The order of the “on” and “off” conditions was randomly assigned and counterbalanced across participants.

An eye-tracker (ASL E-5000) was used to make sure participants did not fall asleep or close their eyes to avoid looking at the visual stimuli. The participants used a plastic box with four push buttons to register their responses.

In a written post-scan questionnaire, participants rated emotions they may have experienced during the interrogation (e.g., upset, anxious, nervous); whether they felt guilty about stealing the object in question; their confidence in getting away with lying (and whether this differed when the lie-detector was active or not), and their motivation to keep the stolen object. Participants responded using a 0–5 scale where 0 means “not at all” and 5 means “a lot.” Additionally, participants were asked whether they had tried to use any strategy to deceive the interrogator, and if they had, to describe this strategy.

### fMRI scanning and preprocessing

A 1.5 Tesla Siemens Sonata MRI scanner (Siemens, Erlangen, Germany) was used to acquire T1-weighted anatomical images and T2^*^-weighted echo-planar functional images with blood oxygenation level-dependent (BOLD) contrast (35 axial slices, 2 mm slice thickness with 1 mm gap, 3 × 3 resolution in plane, slice TE = 50 ms, volume TR = 3.15s, 64 × 64 matrix, 192 × 192mm FOV, 90° flip angle). During two functional EPI sessions, an average of 221 whole brain volumes (range 214–225 depending on participants' response speed) were acquired. The first 4 volumes were discarded to allow for T1 contrast to reach equilibrium.

Image processing was carried out using SPM8 (Statistical Parametric Mapping software, Wellcome Trust Centre for Neuroimaging, UCL; www.fil.ion.ucl.ac.uk/spm) implemented in MATLAB (The Mathworks Inc., Massachusetts, USA; www.mathworks.com). EPI images were realigned to correct for movements by aligning the functional (T2^*^-weighted EPI) images of each run to the first volume using a six-parameter rigid body transformation. Mean functional images were then coregistered to the T1-weighted anatomical image and normalized into Montreal Neurological Institute (MNI) template space using a 12-parameter affine transformation (parameters were estimated from segmentation and normalization of anatomical images to MNI template using SPM8). Normalized functional images were resampled into 2 × 2 × 2 voxel resolution. A Gaussian kernel with a full width at half maximum of 6 mm was applied for spatial smoothing.

### fMRI analysis

In a statistical model that included all events in the scanning run, each event was convolved with the standard haemodynamic response function of SPM8 (Holmes and Friston, 1998). The design matrix comprised a column for each experimental condition, with separate events defined by their onset time and duration (based on participants' response times). The fit to the data was estimated for each participant using a general linear model (Friston et al., 1995) with a 128 s high-pass filter, global scaling, and modeling of serial autocorrelations.

Individual T-contrasts related to the different conditions within a factorial design comprising the conditions of interest (2 factors: lie-detector on vs. off, and true vs. false response) were created from the parameter estimates (beta weights). T-contrasts were computed within subjects for the main effects and interaction between belief about whether the lie-detection device was active and the type of response (true or false) to theft-related questions. These were then used in separate second level random effects analyses in order to facilitate inferences about group effects (Friston et al., 1995). Results are reported for clusters with at least 10 voxels and a significance threshold of *p* < 0.001 (uncorrected for multiple comparisons; Wager et al., 2007). Missed trials were modeled by a regressor of no interest in the GLM analysis. All brain loci are reported in MNI coordinates. Anatomical loci were determined using the Wake Forest University PicAtlas and were double checked against the Harvard-Oxford probabilistic atlas using a 50% probability threshold (Desikan et al., 2006).

## Results

### Debriefing

All participants claimed to have been highly motivated to keep the object they took. Interestingly, 14 of the 17 participants chose to take the memory stick rather than the earphones, claiming in debriefing that they found it more appealing; the fact that the choice was not random confirms that the task was engaging and personally relevant.

Eight of the 17 participants reported that they had tried to use strategies to avoid detection. Strategies included attempting to control their breathing, focusing on something else, silently repeating in their heads *I didn't steal anything*, or trying to prolong their response times when giving truthful answers in an attempt to confuse the interrogator (e.g., one participant said “*I would delay giving a response when asked about the object I didn't steal to create confusion*”).

All the participants reported that they found the interrogation realistic (i.e., none of them suspected that the questions they were asked were actually pre-recorded), though unsurprisingly, some of them noted that they would have been more nervous if the interrogation had not taken place in the context of an experiment. The majority of the participants (12 out of 17) reported that they found it easier to lie when they were told that the lie-detector was inactive.

### Behavioral results

**Figure 2:**
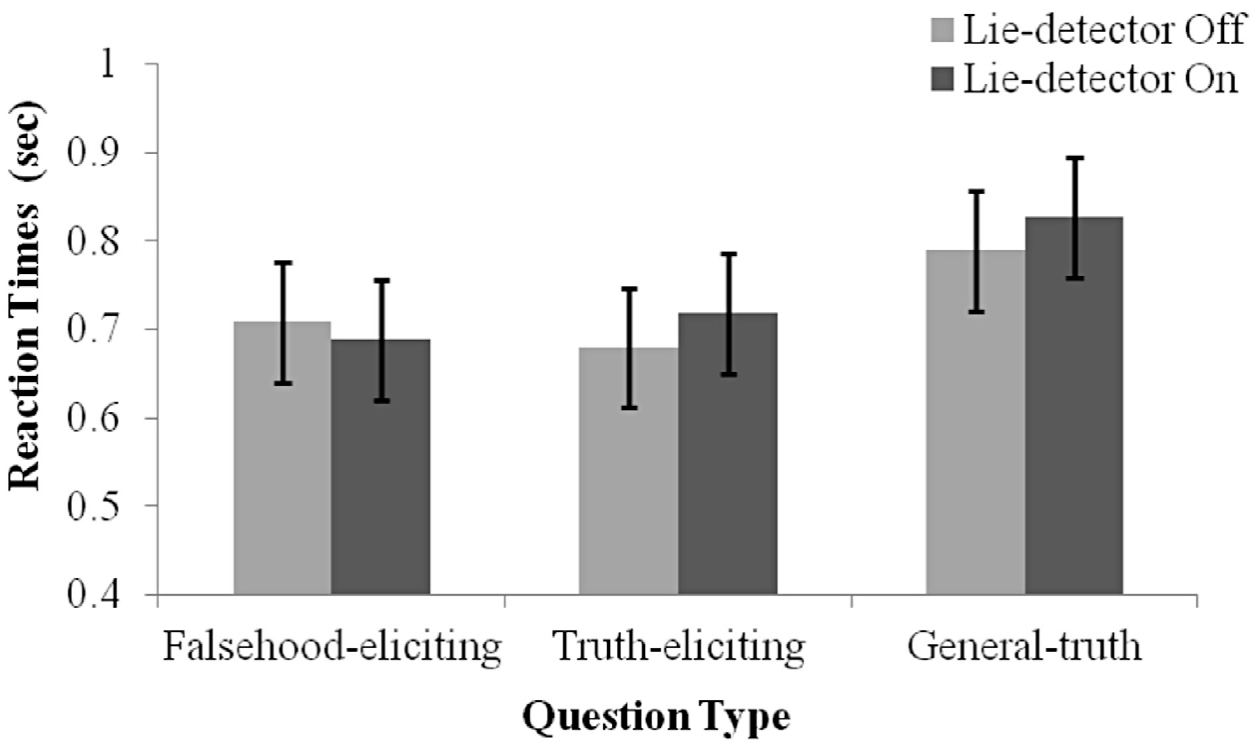
**Mean RT under the different conditions.** Separate means are given for false, true and general responses with the lie-detector “on” and “off”. Error bars represent one standard error of the mean. Participants' responses were slower for general questions than for theft-related questions. RTs to truth- and falsehood-eliciting theft-related questions did not differ, and RTs were not modulated by whether the lie-detector was “on” or “off.”

To examine whether the belief that a “lie-detector” was active affected participants' production of deceptive responses, we examined reaction time (RT) data (Nuñez et al., 2005; Abe et al., 2007; Kozel et al., 2009). RTs were calculated as the duration from the end of a question to the participant's button response. A 2 (belief: “lie-detector” on, “lie-detector” off) × 2 (question type: theft-related truth-eliciting, theft-related falsehood-eliciting) repeated-measures ANOVA revealed no main effects [belief: *F*_(1, 16)_ = 0.169, *p* = 0.69; question type: *F*_(1, 16)_ = 0.00, *p* = 0.97], and no interaction between belief and question type [*F*_(1, 16)_ = 2.381, *p* = 0.142; see Figure 2]. The similarity between the RTs evoked by questions in the different conditions calls into question previous reports (e.g., Nuñez et al., 2005; Abe et al., 2007; Kozel et al., 2009), which suggested that RTs could be used to distinguish deceptive and truthful behavior (but see the Discussion, where we note the limitations of using RTs in the present context).

Interestingly, examination of the general questions indicated that they evoked longer RTs than theft-related ones. Indeed, including them in the statistical analysis, by running a 2 (belief: “Lie-detector” on, “Lie-detector” off) × 3 (question type: theft-related truth-eliciting, theft-related falsehood-eliciting, general truth-eliciting) repeated-measures ANOVA revealed a main effect of question type [*F*_(2, 32)_ = 10.1, *p* < 0.05], but no main effect of belief [*F*_(1, 16)_ = 0.71, *p* = 0.41] nor an interaction between belief and question type [*F*_(2, 32)_ = 1.78, *p* = 0.19] (Figure 2). To investigate the main effect further, *post-hoc* paired *t*-tests [corrected for multiple comparisons using the sequential Bonferroni method (Holm, 1979; Rice, 1989) and collapsed across the belief conditions, as there was no main effect of belief] were conducted. The tests indicated that participants' responses to general questions were slower than to either the theft-related falsehood-eliciting [*t*_(16)_ = 3.45, *p* < 0.05] or theft-related truth-eliciting questions [*t*_(16)_ = 3.31, *p* < 0.05]. RTs to theft-related truth-eliciting and theft-related falsehood-eliciting questions did not differ [*t*_(16)_ = 0.02, *p* = 0.99]. These findings suggest that the increased arousal caused by being asked theft-related questions may have increased the speed with which participants responded to such questions, but the specific content of the questions—whether or not they referred to the object the participant had stolen—did not modulate response times. A different possibility that must be acknowledged is that the pre-recorded theft-related questions were easier to discern while they were still being read out, leading to uniformly faster responses than general questions did.

Three participants explicitly stated in the post-scan questionnaire that they tried to slow their truthful responses in order to mislead the interrogator. However, the behavioral data show that although these three participants made slower responses overall, the patterns of their RTs did not differ from the rest of the group. Despite their claims, their response times were actually slightly faster for true compared to false claims. Excluding these participants did not alter the pattern or significance of any of the analyses reported.

Participants missed an average of 3.62 trials (SD = 4.1) out of a total of 104 trials. One participant missed 14 trials and was the only outlier in terms of missed responses (>3 standard deviations from the mean). This participant's behavioral responses were otherwise within 3 standard deviations from the mean on all measures, and excluding this participant did not alter the pattern or significance of any of the analyses reported.

### Imaging results

To examine the effect of belief on the brain activity underlying the production of deception, we examined BOLD responses evoked by questions in a factorial design with the factors belief (lie-detector on or off) and behavior (true or false responses). Investigations comparing the neural activity associated with true and false responses have been carried out before, and we expected to find increased activation for false (compared to true) responses in similar regions to those found in those previous studies (Ganis et al., 2003; Langleben et al., 2005; Abe et al., 2007; Baumgartner et al., 2009; Kozel et al., 2009; Sip et al., 2012): amygdala, IFG, and PCC. Our main question, however, was whether the difference between the neural activation evoked by false and true responses would be modulated by participants' beliefs about whether their deception could be detected, and whether such modulation would occur in all or only in a subset of the regions that process deception production.

**Figure 3:**
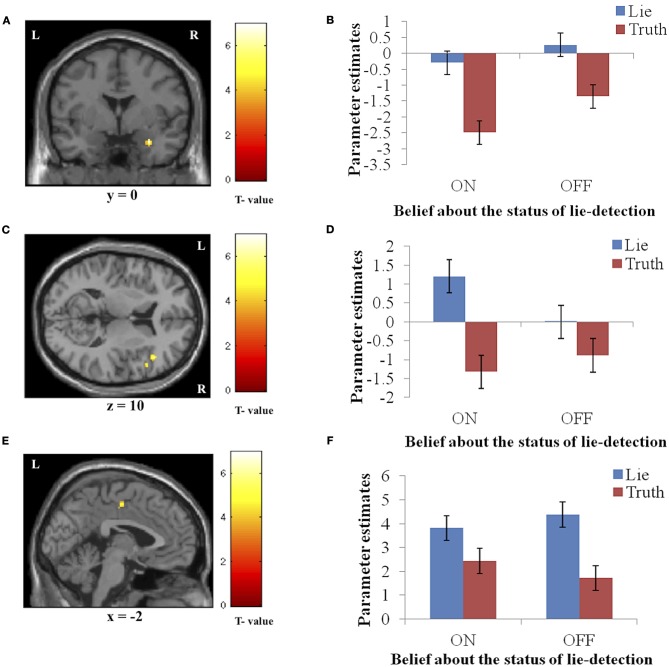
**The main effect of response (false > true).** Panels on the left show the activation cluster and panels on the right show mean parameter estimates in the activation cluster in the right amygdala (**A** and **B**), right IFG (**C** and **D**), and left PCC (**E** and **F**). Deceptive responses in these regions yielded higher BOLD activation than truthful ones, and this difference was not significantly modulated by belief about whether the lie detector was on or off.

Significantly activated regions identified in the second level analysis are detailed in Table 1. The tests revealed a main effect of response type, whereby producing deceptive responses was associated with higher BOLD activation, in the right amygdala and IFG, and in the left PCC (Figure 3). There were no regions in which a main effect in the opposite direction (true > false) was observed, and no regions showed a main effect of belief in either direction (lie-detector on > off or off > on).

**Table 1:** **Brain regions showing activation during response production**.

**Brain region**	**Hemisphere**	***x***	***y***	***z***	***t*-value**	**Cluster size**
**MAIN EFFECT OF RESPONSE (FALSE > TRUE)**						
Amygdala	R	30	0	−24	6.98	17
Inferior frontal gyrus (IFG)	R	44	26	10	6.24	25
Posterior cingulate cortex (PCC)	L	−2	−12	50	4.83	10
**INTERACTION (ON FALSE-TRUE > OFF FALSE-TRUE)**						
Hippocampus/parahippocampal gyrus	R	36	−18	−18	4.98	26
Temporal pole	L	−44	14	−22	4.89	17

Peak activation coordinates in standard MNI space and their associated t-scores. Regions shown were significantly activated at a threshold of p < 0.001 (uncorrected) with a cluster extent threshold of 10 voxels.

**Figure 4:**
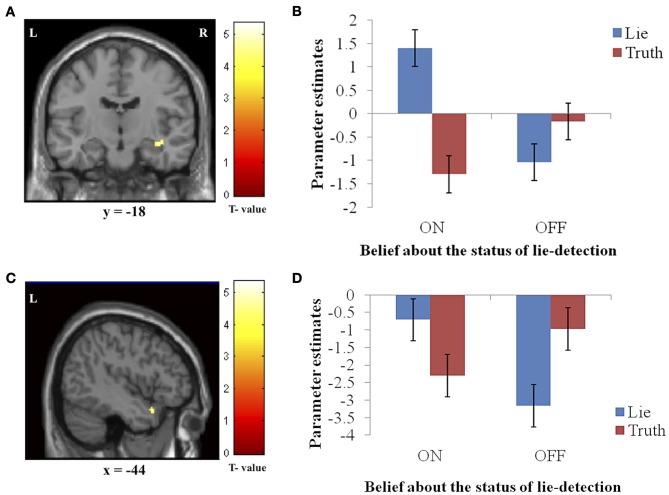
**The interaction between response type (true or false) and belief about the lie-detector (on or off).** Panels on the left show the activation cluster and panels on the right show parameter estimates in the activation cluster in the right hippocampus/parahippocampal gyrus (**A** and **B**) and left temporal pole (**C** and **D**). In these loci, the difference between the BOLD activation caused by false vs. true responses was abolished (and for the hippocampus/parahippocampal gyrus, reversed) when participants believed the lie detector was off.

In addition to the main effects reported above, we found a significant interaction between belief and behavior in two regions: the right hippocampus/parahippocampal gyrus (Figures 4A,B) and the left temporal pole (Figures 4C,D), regions that have both been previously associated with social processes such as theory of mind and face recognition (Olson et al., 2007), and deceptive decision-making (Ganis et al., 2003; Mohamed et al., 2006). Examination of the patterns of responses in these regions reveals that the interaction was due to greater activation when producing deceptive, compared to truthful, responses when the lie-detector was believed to be on, and a reversed pattern when the lie-detector was believed to be off.

To further investigate the effects underlying the interaction, we analyzed the BOLD responses associated with the simple effects of deceptive vs. truthful responses in each belief condition. For the right hippocampus/parahippocampal gyrus, we found that when participants believed the lie-detector was on, activation when producing deceptive responses was significantly greater than when producing truthful responses [*t*_(16)_ = 5.397, *p* < 0.001]. However, when the lie-detector was believed to be off both kinds of response were reduced and were not significantly different from each other [*t*_(16)_ = 1.6, *p* = 0.14]. Belief that lies could be detected thus led to differential responses in this region. For the left temporal pole, there was again significantly greater activation when producing deceptive (compared to truthful) responses in the “lie-detector on” condition [*t*_(16)_ = 2.54, *p* < 0.05]. However, this difference was reversed in the “lie-detector off” condition, in which truthful responses led to significantly greater responses than deceptive responses [*t*_(16)_ = 3.643, *p* < 0.01].

## Discussion

We conducted an fMRI investigation to test whether beliefs about how detectable deception was would affect the neural activity involved in producing it. Specifically, we studied the effect of a belief that a lie-detector was on or off on the neural processing underlying deception. Our results show that a belief in the assumed efficacy of lie-detection does indeed modulate the neural activity in a subset of the regions—the right hippocampus/parahippocampal gyrus and left temporal pole—involved in producing false claims, such that false responses led to greater activity than true responses when participants believed lie-detector was inactive. This difference was not present (and in the left temporal pole was reversed) when the lie-detector was believed to be off. Other regions—right amygdala, right IFG, and left PCC—were more active when producing a false claim, but this difference was not modulated by belief in lie-detectability.

Replicating our previous findings (Sip et al., 2010, 2012), our behavioral results showed that in an ecologically valid scenario there was no difference in RTs for producing true and false statements in a context in which both can be used deceptively. These findings are at odds with other deception studies which have found faster responses when participants were being truthful, compared to when they were producing a false claim (e.g., Kozel et al., 2005; Langleben et al., 2005; Spence et al., 2008; Seymour and Fraynt, 2009). This discrepancy, however, might stem from the realistic experimental paradigm we employed, which may have encouraged some of the participants to attempt to use strategies that would mislead the interrogator. Indeed, during debriefing we learned that some participants had tried to use response timing as a countermeasure to detection. This suggests that people produce deception in various ways if they are allowed to use their own deceptive strategy. However, it must also be noted that in the current study, we used auditory questions combined with visual presentation of relevant items. The visual stimuli may have interfered with auditory processing, or facilitated response preparation such that participants could have decided what response to provide before the question was fully articulated. However, the actual response could only be provided after the question was posed, so calculating RTs as the elapsed time from the end of the question was the only way to avoid additional assumptions regarding the point in time at which participants decided what answer to give. This calculation also avoided false-positive difference in RTs that might be caused by differences in the lengths of the posed questions.

Our neuroimaging results demonstrate that the assumption that the same brain regions would always be either active or inactive when one tells a lie or the truth, respectively (Mohamed et al., 2006) is an oversimplification. Neural activity in various regions, including the ACC, DLPFC, IFG, the caudate nucleus, and the amygdala (e.g., Kozel et al., 2005; Baumgartner et al., 2009; Greene and Paxton, 2009; Sip et al., 2010, 2012; Gamer et al., 2012) has been implicated in the production of deception. The present findings involve a smaller set of areas than reported in previous neuroimaging studies of deception [for a review see Sip et al. (2008a)]. Unlike these previous studies, we did not observe activation in dorsolateral prefrontal cortex (DLPFC), ACC, or the caudate nucleus. The fact that we found activation in a smaller set of regions than previously reported could be due to several factors that are not substantive to the issue of deception, such as the specific statistical model and significance thresholds employed in different studies, specific characteristics of the participant cohort, or the visual and auditory stimuli used in the course of the interrogation. We speculate, however, that a substantive factor—the realistic nature of the mock-theft scenario used in the present study—might also potentially be at play. Such scenarios have been shown previously to reduce participants' physiological arousal (indicated by skin conductance) during interrogation, compared to more standard experimental procedures (though it must be noted that this was observed in the context of a different method for lie-detection, and may have been modulated by reduced memory for crime-related items; Carmel et al., 2003). Although negative findings (the absence of activation in particular brain regions) must always be interpreted with extreme caution, further work may benefit from attempting to address the relation between how realistic a mock-crime scenario is and how widespread neural activation across the brain is during interrogation.

### Main effects: deceptive vs. truthful responses

Deceptive responses produced greater BOLD responses than truthful responses, regardless of the belief condition, in three regions: the right amygdala, right IFG, and left PCC. The amygdala and IFG have been implicated in recent ecologically valid examinations of deception (Abe et al., 2007; Baumgartner et al., 2009; Sip et al., 2012). Here, the observed activation in the amygdala, which is known to be involved in processing emotionally relevant information [for a review see Dolan (2007); Olson et al. (2007)], suggests that participants experienced an emotional conflict resulting from making false claims while risking a potential confrontation, and that this occurred regardless the believed status of the lie-detector device. Abe et al. (2007) were the first to report amygdala involvement in producing verbal deception, employing a realistic scenario in which participants underwent interrogation. They speculated that emotional processing, reflected in the increased amygdala activation they observed, was associated with attempts to deceive the interrogator. In a different study, Baumgartner et al. (2009) showed that breaking a previously expressed promise and consequently deceiving others in a social context appears to create anxiety associated with social consequences of the act rather than with producing false claims *per se.*

In previous studies, the PCC has been implicated in processing the emotional aspects of context and in integrating emotion- and memory-related processes (Mohamed et al., 2006). Here we observe increased activation for producing false vs. true claims, suggesting that the cognitive load associated with deception places demands on emotional processing. This specific processing, however, was not modulated by belief in lie-detectability, indicating that it is largely independent of those processes that mediate the emotion and anxiety engendered by the context of such belief. Previous studies have also shown right IFG involvement in deception (Gamer et al., 2007; Sip et al., 2012) as well as in response inhibition (Aron et al., 2004) and risk aversion (Christopoulos et al., 2009). Interestingly, right IFG was previously involved in production of deceptive responses in a social context where participants had to first comprehend the question, and then choose to whether to inhibit a true response and claim falsehood instead (Sip et al., 2012). The present findings thus suggest that the right IFG plays a generalized role in deception that is related to monitoring response release, and that this process is unlikely to be modulated by belief about lie-detectability.

### Interaction of deceptive/truthful response and belief about lie-detectability

We found two regions, the right hippocampus/parahippocampal gyrus and left temporal pole, in which response and belief interacted significantly to produce greater BOLD activation for deceptive responses when the lie-detector was believed to be on, but not when it was believed to be off. The temporal pole has been implicated in various socio-emotional processes involved in broadly construed theory of mind (Carr et al., 2003; Frith and Frith, 2003; Völlm et al., 2006), moral judgments (Moll et al., 2002; Heekeren et al., 2003), and deception detection (Grèzes et al., 2004, 2006). Olson et al. (2007) suggested that this region thus combines emotional responses with highly processed sensory stimuli. In our study, the increased temporal pole activity we observed when the lie-detector was “on” may be due to participants attempting not only to regulate their own emotional responses but also to infer the emotional states and beliefs of their interrogator. The realistic interrogation scenario, involving an ostensible “real-life interrogator,” may have increased participants' anxiety and contributed to the modulation found in the activity of this region (which is known to have reciprocal anatomical connections to the amygdala; Dolan, 2007; Olson et al., 2007). The pattern of responses in the temporal pole was reversed in the “lie-detector off” condition, in which truthful responses led to greater activation than deceptive ones. The functional significance of this reversal remains unclear and requires further elucidation.

We also observed a differential activation pattern in the right hippocampus/parahippocampal gyrus, where BOLD activity differed for deceptive and truthful responses (and was greater for deceptive ones), but only when the lie-detector was believed to be on. We had not originally included these areas amongst those in which we expected to find differential activation—although the hippocampus has been previously associated with producing deceptive response (Mohamed et al., 2006), and the parahippocampal gyrus has been associated with reporting autobiographical memories (which participants must draw on to produce truthful and deceptive responses; Ganis et al., 2003), neither region has been reported as consistently as other regions in the context of deception [for an overview, see e.g., Sip et al. (2008a)]. The differential activation we find here suggests that these areas may play a role related to belief, which had not been tapped into by previous studies where this factor was not manipulated.

The hippocampus is known to play a central role in memory (e.g., Burgess et al., 2002) as well as predictions about upcoming events related to past experiences [for a review see e.g., Buckner (2010)]. A previous investigation of neural connectivity (Smith et al., 2006) has shown that not only the content of a memory but also the context in which a memory was created have a measurable impact on episodic retrieval and interpersonal communication. It is thus noteworthy that the cluster of activation that included the hippocampus also extended to the parahippocampal gyrus. In a social context the parahippocampal gyrus (as well as temporal pole) allows for a proper identification of communicational intent, as demonstrated in a previous study of sarcasm (Rankin et al., 2009). A seemingly insincere communication, such as sarcasm, shares certain characteristics with deception, as in both the communicated content is at odds with reality. However, in contrast to deception, sarcasm lacks the deceptive intent; listener is meant to realize the true meaning of what is communicated. Importantly, to distinguish sarcasm from deception, one needs to identify the meaning based on contextual cues. Similarly, in the current study, participants interacted with another person and based on prosody cues obtained from the interrogator, had to monitor whether their denial of an action they did remember performing (e.g., stealing a pair of ear phones) could be successful. Their belief regarding whether the lie-detector is active was thus directly relevant to this process of inference. The right parahippocampal gyrus may therefore perform a similar role, mediating social interaction, and its underlying intent, in both the contextualized production of deception of the present study and in processing sarcasm (Rankin et al., 2009).

Emotionally charged experiences involve the hippocampus, parahippocampal gyrus, and amygdala in the process of encoding and consolidating these events into memories (Richter-Levin and Akirav, 2001). The hippocampus is known to play a crucial role in associative learning, as well as encoding and representing the value of reward (e.g., Richter-Levin and Akirav, 2001; Smith et al., 2006; Wimmer and Shohamy, 2012). Recently, Wimmer and Shohamy (2012) offered novel neural evidence indicating that the hippocampus may play an important role in value-based decision-making. They showed that the hippocampus not only encodes reward value but also spreads it across items that were not previously considered rewarding. In light of the present findings, we propose that the neural connectivity between the hippocampus/parahippocampal gyrus and amygdala (Phelps, 2004; Smith et al., 2006) may facilitate a similar role for the hippocampus/parahippocampal gyrus in context-dependent social interactions, where social value must be flexibly assigned.

Interestingly, although activity in the amygdala was significantly modulated by response (deceptive vs. truthful), this modulation did not interact with belief about the status of the lie-detector. Our original hypothesis that the amygdala would be a prime candidate for belief-related modulation was therefore not borne out. Importantly, previous studies reporting deception-related amygdala activation (Abe et al., 2007; Baumgartner et al., 2009) did not have the immediate confrontation element that was present in the interrogation scenario of the current study. The absence of a significant interaction in the amygdala could thus be due either to belief modulating other functions than the emotional processes associated with amygdala activity, or to a ceiling effect—the interrogation context may have been sufficient to induce differential deception-related activity regardless of belief about lie-detectability.

## Conclusions

Overall, our findings suggest that belief in lie-detection efficacy modulates a subset of the processes involved in producing deception. Cognitive processes involving reasoning and theory of mind, mediated by the IFG and PCC, as well as emotional processes mediated by the amygdala, are involved in the production of deception—but the absence of modulation by belief in these regions suggests that the processes they mediate are functionally separate from those involving belief. However, belief about the detectability of lies does modulate activity in the temporal pole and hippocampus/parahippocampal gyrus, suggesting that the social context and memory-related processing known to be mediated by these regions are the aspects of deception that are affected by belief. We, therefore, conclude that belief in the efficacy of a lie-detection device matters, emphasizing the importance of such beliefs in both basic research and applied (forensic) settings.

### Conflict of interest statement

The authors declare that the research was conducted in the absence of any commercial or financial relationships that could be construed as a potential conflict of interest.

## References

[B1] Abe N. Suzuki M. Mori E. Itoh M. Fujii T. (2007). Deceiving others: distinct neural responses of the prefrontal cortex and amygdala in simple fabrication and deception with social interactions. J. Cogn. Neurosci. 19, 287–295 10.1162/jocn.2007.19.2.287 17280517

[B2] Aron A. R. Robbins T. W. Poldrack R. A. (2004). Inhibition and the right inferior frontal cortex. Trends Cogn. Sci. 8, 170–177 10.1016/j.tics.2004.02.010 15050513

[B3] Baumgartner T. Fischbacher U. Feierabend A. Lutz K. Fehr E. (2009). The neural circuitry of a broken promise. Neuron 64, 756–770 10.1016/j.neuron.2009.11.017 20005830

[B3a] Bell B. G. Kircher J. C. Bernhardt P. C. (2008). New measures improve the accuracy of the directed-lie test when detecting deception using a mock crime. Physiol. Behav. 94, 331–340 10.1016/j.physbeh.2008.01.022 18343464

[B4] Buckner R. L. (2010). The role of the hippocampus in prediction and imagination. Annu. Rev. Psychol. 61, 27–48 10.1146/annurev.psych.60.110707.163508 19958178

[B5] Burgess N. Maguire E. A. O'Keefe J. (2002). The human hippocampus and spatial and episodic memory. Neuron 35, 625–641 10.1016/S0896-6273(02)00830-9 12194864

[B6] Carmel D. Dayan Y. Naveh A. Raveh O. Ben-Shakhar G. (2003). Estimating the validity of the guilty knowledge test from simulated experiments: the external validity of mock crime studies. J. Exp. Psychol. Appl. 9, 261–269 10.1037/1076-898X.9.4.261 14664677

[B7] Carr L. Iacoboni M. Dubeau M. C. Mazziotta J. C. Lenzi G. L. (2003). Neural mechanisms of empathy in humans: a relay from neural systems for imitation to limbic areas. Proc. Natl. Acad. Sci. U.S.A. 100, 5497–5502 10.1073/pnas.0935845100 12682281 PMC154373

[B8] Christopoulos G. I. Tobler P. N. Bossaerts P. Dolan R. J. Schultz W. (2009). Neural correlates of value, risk, and risk aversion contributing to decision making under risk. J. Neurosci. 26, 6469–6472 10.1523/JNEUROSCI.2614-09.2009 19812332 PMC2794196

[B9a] De Martino B. Kumaran D. Seymour B. Dolan R. J. (2006). Frames, biases, and rational decision-making in the human brain. Science 313, 684–687 10.1126/science.1128356 16888142 PMC2631940

[B9] Desikan R. S. Ségonne F. Fischl B. Quinn B. T. Dickerson B. C. Blacker D. (2006). An automated labeling system for subdividing the human cerebral cortex on MRI scans into gyral based regions of interest. Neuroimage 31, 968–980 10.1016/j.neuroimage.2006.01.021 16530430

[B10] Dolan R. J. (2007). The human amygdala and orbital prefrontal cortex in behavioural regulation. Philos. Trans. R. Soc. Lond. B Biol. Sci. 362, 787–799 10.1098/rstb.2007.2088 17403643 PMC2429997

[B11] Ekman P. (1992). Telling Lies: Clues to Deceit in the Marketplace, Politics and Marriage. New York, NY: W W Norton

[B12] Ekman P. Friesen W. V. (1969). Nonverbal leakage and clues to deception. Psychiatry 32, 88–106 5779090 10.1080/00332747.1969.11023575

[B13] Friston K. J. Holmes A. P. Poline J. B. Grasby P. J. Williams S. C. Frackowiak R. S. (1995). Analysis of fMRI time-series revisited. Neuroimage 2, 45–53 10.1006/nimg.1995.1007 9343589

[B14] Frith U. Frith C. D. (2003). Development and neurophysiology of mentalizing. Philos. Trans. R. Soc. Lond. B Biol. Sci. 358, 459–473 10.1098/rstb.2002.1218 12689373 PMC1693139

[B15] Gamer M. Bauermann T. Stoeter P. Vossel G. (2007). Covariations among fMRI, skin conductance, and behavioral data during processing of concealed information. Hum. Brain Mapp. 28, 1287–1301 10.1002/hbm.20343 17290371 PMC6871443

[B17] Gamer M. Klimecki O. Bauermann T. Stoeter P. Vossel G. (2012). fMRI-activation patterns in the detection of concealed information rely on memory-related effects. Soc. Cogn. Affect. Neurosci. 7, 506–515 10.1093/scan/nsp005 19258375 PMC3375883

[B16] Gamer M. Kosiol D. Vossel G. (2010). Strength of memory encoding affects physiological responses in the Guilty Actions Test. Biol. Psychol. 83, 101–107 10.1016/j.biopsycho.2009.11.005 19931347

[B18] Ganis G. Kosslyn S. M. Stose S. Thompson W. L. Yurgelun-Todd D. A. (2003). Neural correlates of different types of deception: an fMRI investigation. Cereb. Cortex 13, 830–836 10.1093/cercor/13.8.830 12853369

[B19] Greely H. T. Illes J. (2007). Neuroscience-based lie detection: the urgent need for regulation. Am. J. Law Med. 33, 377–431 17910165 10.1177/009885880703300211

[B19a] Greene J. D. Paxton J. M. (2009). Patterns of neural activity associated with honest and dishonest moral decisions. Proc. Natl. Acad. Sci. U.S.A. 106, 12506–12511 10.1073/pnas.0900152106 19622733 PMC2718383

[B20] Grèzes J. Berthoz S. Passingham R. E. (2006). Amygdala activation when one is the target of deceit: did he lie to you or to someone else? Neuroimage 30, 601–608 10.1016/j.neuroimage.2005.09.038 16257239

[B21] Grèzes J. Frith C. D. Passingham R. E. (2004). Brain mechanisms for inferring deceit in the actions of others. J. Neurosci. 24, 5500–5505 10.1523/JNEUROSCI.0219-04.2004 15201322 PMC6729335

[B22] Heekeren H. R. Wartenburger I. Schmidt H. Schwintowski H. P. Villringer A. (2003). An fMRI study of simple ethical decision-making. Neuroreport 14, 1215–1219 10.1097/01.wnr.0000081878.45938.a7 12824762

[B23] Holm S. (1979). A simple sequentially rejective multiple test procedure. Scand. J. Stat. 6, 65–70

[B24] Holmes A. P. Friston K. J. (1998). Generalisability, random effects and population inference. Neuroimage 7, 754

[B25] Kozel F. A. Johnson K. A. Grenesko E. L. Laken S. J. Kose S. Lu X. (2009). Functional MRI detection of deception after committing a mock sabotage crime. J. Forensic Sci. 54, 220–231 10.1111/j.1556-4029.2008.00927.x 19067772 PMC2735094

[B26] Kozel F. A. Johnson K. A. Mu Q. Grenesko E. L. Laken S. J. George M. S. (2005). Detecting deception using functional magnetic resonance imaging. Biol. Psychiatry 58, 605–613 10.1016/j.biopsych.2005.07.040 16185668

[B27] Langleben D. D. Loughead J. W. Bilker W. B. Ruparel K. Childress A. R. Busch S. I. (2005). Telling truth from lie in individual subjects with fast event-related fMRI. Hum. Brain Mapp. 26, 262–272 10.1002/hbm.20191 16161128 PMC6871667

[B28] Mobbs D. Weiskopf N. Lau H. C. Eric Featherstone E. Dolan R. J. Frith C. D. (2006). The kuleshov effect: the influence of contextual framing on emotional attributions. Soc. Cogn. Affect. Neurosci. 1, 95–106 10.1093/scan/nsl014 17339967 PMC1810228

[B29] Mohamed F. B. Faro S. H. Gordon N. J. Platek S. M. Ahmad H. Williams J. M. (2006). Brain mapping of deception and truth telling about an ecologically valid situation: functional MR imaging and polygraph investigation–initial experience. Radiology 238, 679–688 10.1148/radiol.2382050237 16436822

[B30] Moll J. de Oliveira-Souza R. Eslinger P. J. Bramati I. E. Mourao-Miranda J. Andreiuolo P. A. (2002). The neural correlates of moral sensitivity: a functional magnetic resonance imaging investigation of basic and moral emotions. J. Neurosci. 22, 2730–2736 11923438 10.1523/JNEUROSCI.22-07-02730.2002 PMC6758288

[B31a] Nuñez J. M. Casey B. J. Egner T. Hare T. Hirsch J. (2005). Intentional false responding shares neural substrates with response conflict and cognitive control. Neuroimage 25, 267–277 10.1016/j.neuroimage.2004.10.041 15734361

[B31] Olson I. R. Plotzker A. Ezzyat Y. (2007). The Enigmatic temporal pole: a review of findings on social and emotional processing. Brain 130, 1718–1731 10.1093/brain/awm052 17392317

[B32] Petrovic P. Dietrich T. Fransson P. Andersson J. Carlsson K. Ingvar M. (2005). Placebo in emotional processing– induced expectations on anxiety relief activate a generalized modulatory network. Neuron 46, 957–969 10.1016/j.neuron.2005.05.023 15953423

[B33] Phelps E. A. (2004). Human emotion and memory: interactions of the amygdala and hippocampal complex. Curr. Opin. Neurobiol. 14, 198–202 10.1016/j.conb.2004.03.015 15082325

[B34] Pollina D. A. Dollins A. B. Senter S. M. Krapohl D. J. Ryan A. H. (2004). Comparison of polygraph data obtained from individuals involved in mock crimes and actual criminal investigations. J. Appl. Psychol. 89, 1099–1105 10.1037/0021-9010.89.6.1099 15584845

[B35] Rankin K. P. Salazar A. Gorno-Tempini M. L. Sollberger M. Wilson S. M. Pavlic D. (2009). Detecting sarcasm from paralinguistic cues: anatomic and cognitive correlates in neurodegenerative disease. Neuroimage 47, 2005–2015 10.1016/j.neuroimage.2009.05.077 19501175 PMC2720152

[B36] Rice W. R. (1989). Analyzing tables of statistical tests. Evolution 6, 223–225 10.1111/j.1558-5646.1989.tb04220.x 28568501

[B37] Richter-Levin G. Akirav I. (2001). Amygdala-hippocampus dynamic interaction in relation to memory. Mol. Neurobiol. 22, 11–20 11414274 10.1385/MN:22:1-3:011

[B38] Seymour T. L. Fraynt B. R. (2009). Time and encoding effects in the concealed knowledge test. Appl. Psychophysiol. Biofeedback 34, 177–187 10.1007/s10484-009-9092-3 19536648 PMC2727398

[B39] Sip K. E. Lynge M. Wallentin M. McGregor W. B. Frith C. D. Roepstorff A. (2010). The production and detection of deception in an interactive game. Neuropsychologia 48, 3619–3626 10.1016/j.neuropsychologia.2010.08.013 20727906

[B40] Sip K. E. Roepstorff A. McGregor W. B. Frith C. D. (2008a). Detecting deception: the scope and limits. Trends Cogn. Sci. 12, 48–53 10.1016/j.tics.2007.11.008 18178516

[B41] Sip K. E. Roepstorff A. McGregor W. B. Frith C. D. (2008b). Response to Haynes: there's more to deception than brain activity. Trends Cogn. Sci. 12, 126–127 18178516 10.1016/j.tics.2007.11.008

[B42] Sip K. E. Skewes J. C. Marchant J. L. McGregor W. B. Roepstorff A. Frith C. D. (2012). What if I get busted? Deception, choice, and decision-making in social interaction. Front. Neurosci. 6:58 10.3389/fnins.2012.00058 22529772 PMC3328780

[B43] Smith A. P. Stephan K. E. Rugg M. D. Dolan R. J. (2006). Task and content modulate amygdala-hippocampal connectivity in emotional retrieval. Neuron 49, 631–638 10.1016/j.neuron.2005.12.025 16476670

[B44] Spence S. A. Farrow T. F. D. Herford A. E. Wilkinson I. D. Zheng Y. Woodruff P. W. R. (2001). Behavioural and functional anatomical correlates of deception in humans. Neuroreport 12, 2849–2853 11588589 10.1097/00001756-200109170-00019

[B45] Spence S. A. Kaylor-Hughes C. Farrow T. F. D. Wilkinson I. D. (2008). Speaking of secrets and lies: the contribution of ventrolateral prefrontal cortex to vocal deception. Neuroimage 40, 1411–1418 10.1016/j.neuroimage.2008.01.035 18308586

[B46] Völlm B. A. Taylor A. N. W. Richardson P. Rhiannon C. Stirling J. McKie S. (2006). Neuronal correlates of theory of mind and empathy: a functional magnetic resonance imaging study in a nonverbal task. Neuroimage 29, 90–98 10.1016/j.neuroimage.2005.07.022 16122944

[B47c] Vrij A. (1993). Credibility judgments of detectives: the impact of nonverbal behavior, social skills, and physical characteristics on impression formation. J. Soc. Psychol. 133, 601–610 10.1080/00224545.1993.9713915 8283858

[B47d] Vrij A. (2000). Detection Lies and Deceit. The Psychology of Lying and the Implications for Professional Practice. Chichester: John Wiley & Sons, Ltd

[B47e] Vrij A. (2004). Why professionals fail to catch liars and how they can improve. Leg. Criminol. Psychol. 9, 159–181

[B47f] Vrij A. (2008). Nonverbal dominance versus verbal accuracy in lie detection. Crim. Just. Behav. 35, 1323–1336

[B47a] Vrij A. Edward K. Bull R. (2001). People's insight into their own behaviour and speech content while lying. Br. J. Psychol. 92, (Pt 2), 373–389 11417787

[B47b] Vrij A. Mann S. Kristen S. Fisher R. P. (2007). Cues to deception and ability to detect lies as a function of police interview styles. Law Hum. Behav. 31, 499–518 10.1007/s10979-006-9066-4 17211691

[B47] Wager T. D. Lindguist M. Kaplan L. (2007). Meta-analysis of functional neuroimaging data: current and future directions. Soc. Cogn. Affect. Neurosci. 2, 150–158 10.1093/scan/nsm015 18985131 PMC2555451

[B48] Wimmer G. E. Shohamy D. (2012). Preference by association: how memory mechanisms in the hippocampus bias decisions. Science 338, 270–273 10.1126/science.1223252 23066083

